# Advances in the development of tubular structures using extrusion-based 3D cell-printing technology for vascular tissue regenerative applications

**DOI:** 10.1186/s40824-022-00321-2

**Published:** 2022-12-05

**Authors:** Gi Hoon Yang, Donggu Kang, SangHyun An, Jeong Yeop Ryu, KyoungHo Lee, Jun Sik Kim, Moon-Yong Song, Young-Sik Kim, Sang-Mo Kwon, Won-Kyo Jung, Woonhyeok Jeong, Hojun Jeon

**Affiliations:** 1 Research Institute of Additive Manufacturing and Regenerative Medicine, Baobab Healthcare Inc, 55 Hanyangdaehak-Ro, Ansan, Gyeonggi-Do 15588 South Korea; 2 Preclinical Research Center, K Medi-hub, 80 Cheombok-ro, Dong-gu, Daegu, 41061 South Korea; 3 Department of Plastic and Reconstructive Surgery, School of Medicine, Kyungpook National University, 130 Dongdeok‑ro, Jung‑gu, Daegu, 41944 South Korea; 4 Medical Safety Center, Bio Division, Korea Conformity Laboratories 8, Gaetbeol-ro 145beon-gil, Yeonsu-gu, Incheon, 21999 South Korea; 5 Department of Physiology, School of Medicine, Laboratory for Vascular Medicine and Stem Cell Biology, Medical Research Institute, Immunoregulatory Therapeutics Group in Brain Busan 21 Project, Pusan National University, Yangsan, 626-870 South Korea; 6 Division of Biomedical Engineering and Research Center for Marine Integrated Bionics Technology, Pukyong National University, Daeyeon-dong, Nam-gu, Busan, 48513 South Korea; 7 Department of Plastic and Reconstructive Surgery, Dongsan Medical Center, Keimyung University College of Medicine, 1035 Dalgubeol-daero, Dalseo-gu, Daegu, 42601 South Korea

**Keywords:** Small diameter, Vascular grafts, Vascular tissue engineering, 3D cell-printing

## Abstract

Until recent, there are no ideal small diameter vascular grafts available on the market. Most of the commercialized vascular grafts are used for medium to large-sized blood vessels. As a solution, vascular tissue engineering has been introduced and shown promising outcomes. Despite these optimistic results, there are limitations to commercialization. This review will cover the need for extrusion-based 3D cell-printing technique capable of mimicking the natural structure of the blood vessel. First, we will highlight the physiological structure of the blood vessel as well as the requirements for an ideal vascular graft. Then, the essential factors of 3D cell-printing including bioink, and cell-printing system will be discussed. Afterwards, we will mention their applications in the fabrication of tissue engineered vascular grafts. Finally, conclusions and future perspectives will be discussed.

## Introduction

### The need for an ideal small diameter vascular graft

Vascular autografts are widely used in treatment of vascular diseases and in surgeries such as tissue reconstruction and replantation. The first use of autologous artery was reported in 1896 [1]. Up till now, autografts are known as the gold standard of vascular replacement. Although autografts have various benefits including appropriate mechanical properties, the major drawback is the limited availability [2]. Since then, various synthetic vascular grafts were commercialized using Dacron, expanded poly(tetrafluoroethylene) (ePTFE), and polyurethane (PU) (Table 1) [3]. However, these FDA approved nondegradable synthetics may not be the best option due to the risk of thrombosis, intimal hyperplasia, and graft failure in small diameter environments [4–7]. Therefore, new approaches were needed to compensate the limitations of autologous and synthetic vascular grafts. By doing so, vascular tissue engineering (VTE) has emerged to bridge the gap [8–12].

**Table 1:** Examples of commercialized vascular grafts

**Product**	**Company**	**Material**	**Diameter (mm)**	**Ref.**
AlboGraft	LeMaitre Vascular	Polyester (knitted)	6–24	[13]
Gore-Tex	W. L. Gore & Associates	e-PTFE	4–8	[14]
Artegraft	LeMaitre Vascular	Bovine carotid artery	4–8	[15]
Cryovein	CryoLife	Cadaver Saphenous Vein	3–6	[16]
SynerGraft	CryoLife	Decellularized bovine ureter	7	[17]

The purpose of VTE is to develop tissue engineered tubular scaffolds mimicking the in vivo environment of the native blood vessels. One of the most important factors to imitate is the mechanical properties which should match with the host blood vessel. Low mechanical strength may lead to rupture of the graft due to the constant blood pressure, while high stiffness can cause compliance mismatch between the graft and the native blood vessel leading to intimal hyperplasia or atherosclerosis [18, 19]. Besides the mechanical properties, the diameter of the tubular scaffold should match to that of the host blood vessel. Size mismatch between the host and the transplanted vessels can cause uncontrolled turbulence or resistance [20, 21]. Other requirements are that the vascular graft should reduce the risk of thrombogenicity while enhancing the regenerative potentials [22].

**Fig. 1:**
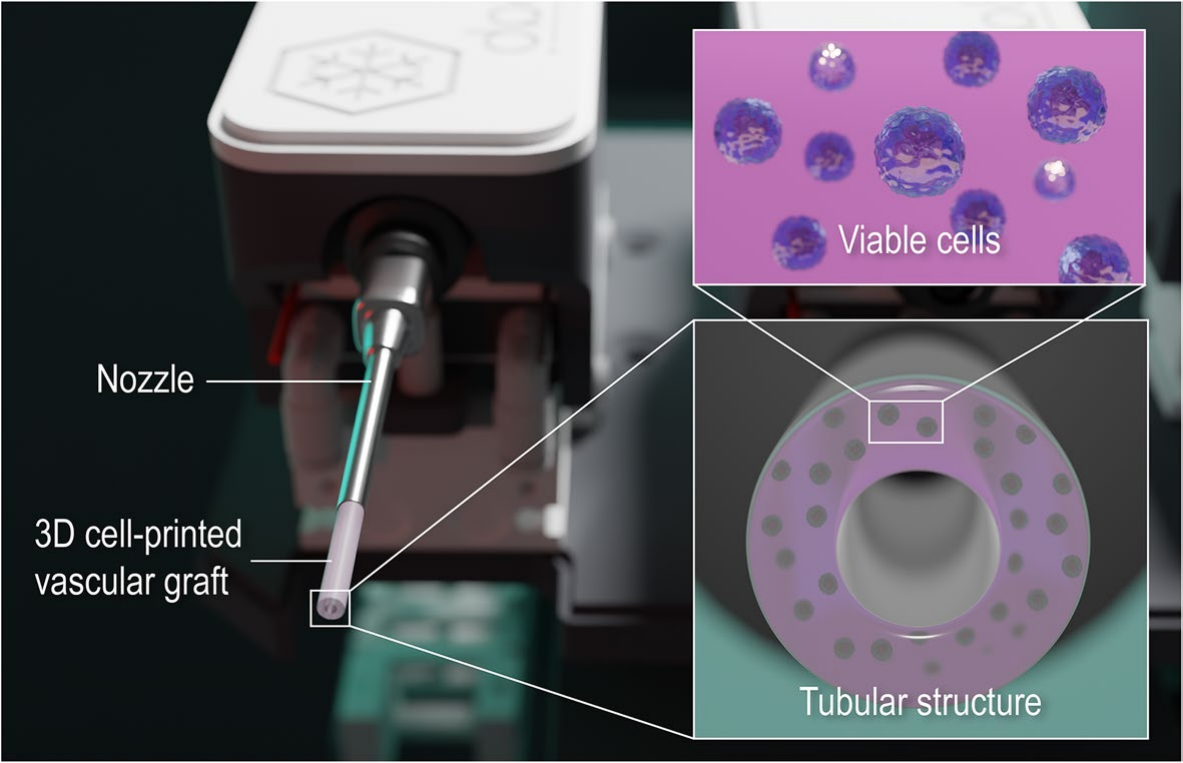
Schematical visualization of a 3D cell-printing process for VTE

Recently, advancements in tissue engineering have enabled mimicry of tissues and organs in a more precise manner. Especially, the introduction of 3D cell-printing allowed the deposition of cells accurately and uniformly in the desired region of the scaffold [23–26]. Through this technique, cell-encapsulated microtubular structures can be fabricated for VTE applications (Fig. 1). This review introduces the current development in tissue engineered vascular scaffolds created using extrusion-based 3D cell-printing technique.

## Blood vessel structure and physiology

Prior to developing tissue engineered blood vessels, understanding of the structure and functions of the native blood vessels is the fundamental step. The native circulation system starts with the outflow of oxygen-rich blood through the aorta which branches into arteries to other organs and tissues. Arteries further branches into arterioles and then divide into capillaries, the smallest blood vessels. The actual interactions between local cells and blood such as nutrient supply and waste removal take place in the capillaries [27]. Then, the deoxygenated blood is collected by venules and further transported to veins which returns to the heart [28].

### Types of the blood vessels and their structures

**Fig. 2:**
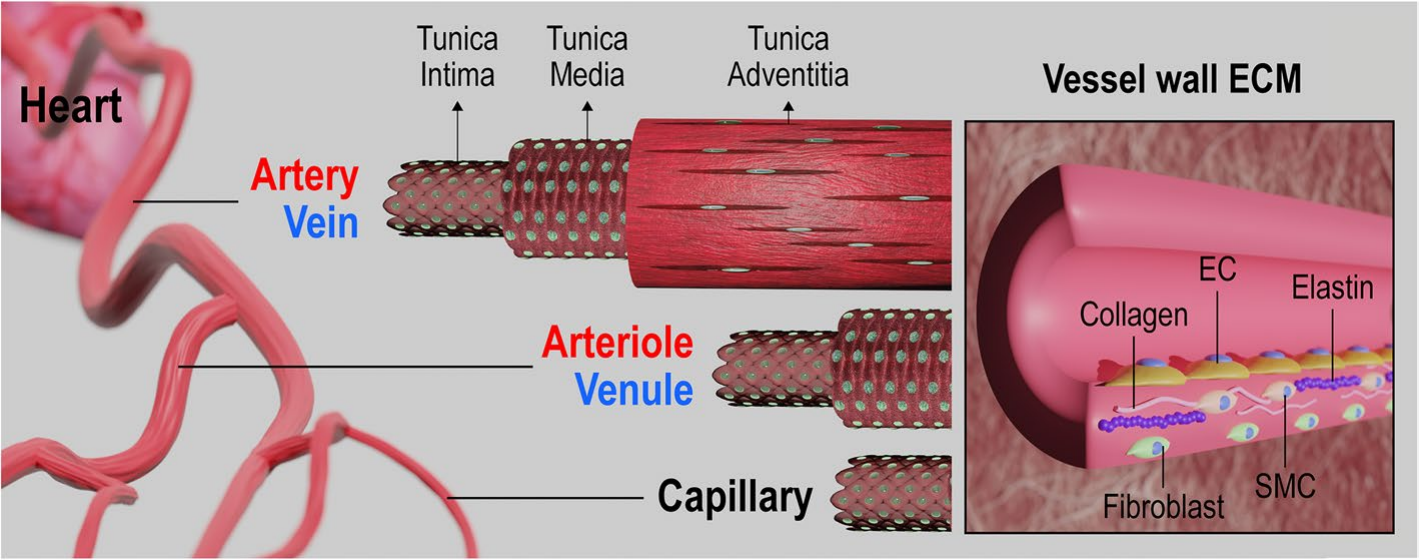
Structure of the artery, vein, and capillary

Arteries and veins are composed of three distinct layers: (1) tunica intima, (2) - media, and (3) - adventitia (Fig. 2). However, the thickness of the layers vary depending on their physiological role [29]. The tunica intima which is the inner layer is made up of endothelial cells (ECs) providing a pathway for frictionless flow of blood. This tight monolayer also functions for antithrombosis, anti-infection or -inflammation, and regulation of cells in other layers by detecting physicochemical and biological changes in the blood [30, 31]. The middle layer or tunica media responsible for integrity and mechanical strength of the blood vessel is composed of smooth muscle cells (SMCs) in circle of rows and elastic fibers [32, 33]. The outer layer, tunica adventitia, consists of fibroblasts, collagen, and elastic fibers forming the connective tissue. Most of the collagen fibrils are circumferentially oriented, while the fibrils on the surface are longitudinally oriented [34, 35]. This composition attributes to passive mechanical support including preventing overexpansion of the vessel [36]. The smaller vessels, arterioles and venules, are composed of two layers which are tunica intima and – media. Finally, capillaries are composed of a thin endothelialized mono-layer where the blood flow is the slowest [31].

### The importance of ECM structure

The two main building blocks of the vessel wall extracellular matrix (ECM) engaged in the mechanical properties are collagen and elastin [37–39]. Collagen is one of the most abundant protein in the body providing framework to tissues and organs [40]. Collagen has a triple-helical structure composed of three polypeptide chains which are held together by hydrogen bonds [41]. Fibrillar collagens are composed of fibers which are bundles of collagen fibrils. These fibrils are aggregates of the precursors called tropocollagen. Elastin is an insoluble hydrophobic protein found in the ECM providing various tissues with elasticity [42]. Elastogenesis is initiated by the expression of tropoelastin, a precursor of elastin, from a single human gene called ELN [43]. Then, these precursors are secreted into the extracellular space by vascular cells including SMCs, ECs, and fibroblasts. After the secretion, the soluble monomers experience a process known as coacervation. The coacervates attached to the cell surface undergo partial crosslinking and detach from the cell membrane. Further crosslinking results in the maturation of elastic fibers [44]. The elasticity and stiffness of the blood vessel is determined by the structure of the ECM. The difference in structure depends on their anatomic location which determine their functional role. For example, compared to veins and venules, the wall thickness of arteries and arterioles are relatively thicker in order to maintain the blood pressure and control the blood flow [45].

In general, the ECM serves as the scaffold providing stability and structural integrity to tissues and organs [46]. Moreover, the ECM allows information exchange with cells for the regulation of various cellular activities [47]. The ECM in the blood vessels attributes to various functions. Most importantly, the vascular ECM is engaged in the mechanical properties of the blood vessel [48]. The blood vessel is capable of bearing the mechanical forces driven by the everflowing blood owing to the ECM.

There are a few factors affecting the wall ECM structure including wall shear and circumferential stress. Wall shear stress is defined as the frictional force per unit area and circumferential or hoop stress is the force acting tangentially to the circumference exerted by the circulating blood flow on the intimal surface of the blood vessel [49, 50]. This can be explained using the Hagen-Poiseuille equation (shear stress ($\tau ) =32\eta Q/\pi {d}^{3}$, where $\eta$ indicates the mean viscosity, $Q$ indicates the mean blood flow rate, and $d$ indicates the vessel diameter) and hoop stress formula (circumferential stress ($\sigma ) = Pd/2w$, where *P* indicates the internal pressure and *w* indicates the wall thickness) [51, 52]. High degree of shear and circumferential stress results in increased vessel wall thickness and diameter to maintain the normal shear stress value [53]. On the contrary, low value of stresses reduces the vessel diameter leading to intimal hyperplasia [53]. Likewise, these environmental changes trigger cellular activities as well as the remodeling of the blood vessel.

## Requirements for vascular grafts

**Fig. 3:**
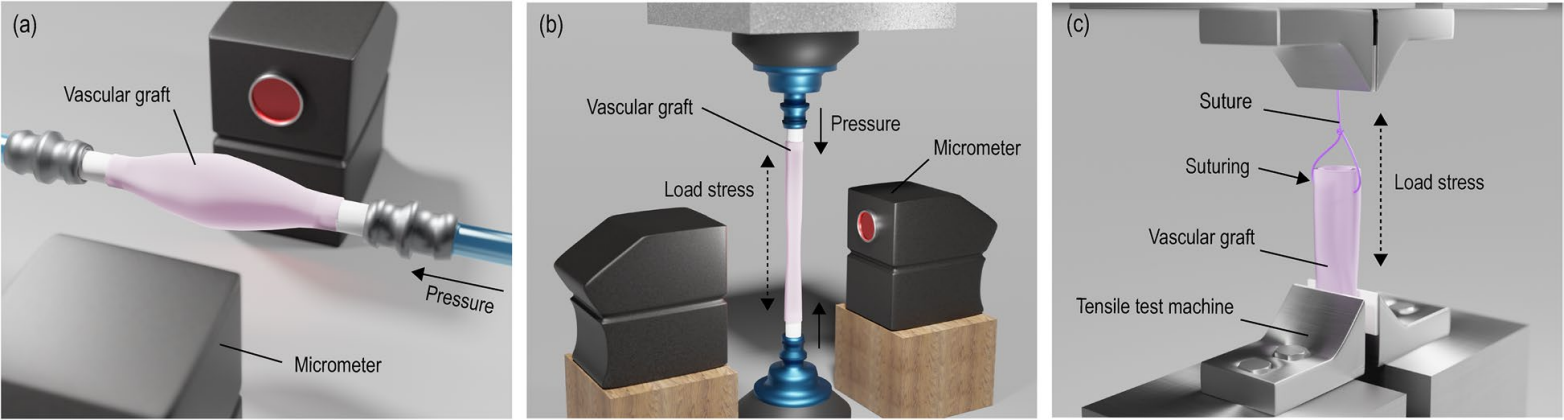
Schematical illustration related to the (**a**) burst, (**b**) compliance, and (**c**) suture retention tests

Until now, there are no obvious guidelines related to the data on the physical and chemical properties and performance of the biodegradable scaffold for vascular regeneration manufactured using a 3D bioprinter. Moreover, vascular graft with cells encapsulated are even more complicated. However, there are guidelines related to the data on the cardiovascular implants - tubular vascular prostheses (ISO 7198:2016) [54]. Typical evaluations performed on vascular grafts are burst pressure, compliance, and suture retention which are related to the mechanical properties (Fig. 3). Besides the physical characteristics, endothelialization is a crucial process in vascular regeneration.

### Burst pressure

Burst pressure is one of the most important parameters since vascular grafts should withstand the hemodynamic pressures. Therefore, the vascular transplant must have sufficient strength to avoid rupture or permanent deformation. The greatest pressure before failure of the graft is termed the burst strength [55]. The burst strength can be calculated using the equation, ${P}_{\text{b}\text{u}\text{r}\text{s}\text{t}}= {\sigma }_{y} \times t/r$, where ${\sigma }_{y}$ defines the yield stress, $t$ defines the wall thickness, and $r$ defines the radius of the vascular graft. This equation shows that the burst pressure increases linearly with decreasing radius assumed that the wall thickness is constant. In general, the burst pressure is measured by pressurizing the vascular graft at 80–120 mmHg s^− 1^ until rupture, while the internal pressure is recorded (Fig. 3(a)). The maximum pressure at rupture is referred to as the burst pressure.

### Compliance

Compliance mismatch between the host blood vessel and vascular graft can cause various side effects including intimal hyperplasia and occlusion due to hemodynamic flow changes across anastomosis [56–58]. Compliance measures the dimensional change of a graft over a change in internal pressure [59]. This can be described using the following equation, $\%\text{c}\text{o}\text{m}\text{p}\text{l}\text{i}\text{a}\text{n}\text{c}\text{e} = \left(\frac{{r}_{{p}_{2}}- {r}_{{p}_{1}}}{{r}_{{p}_{1}}}/{p}_{2} - {p}_{1}\right)\times {10}^{4}$, in which *r* indicates the radius and *p* indicates the pressure. The most commonly used synthetic grafts (Dacron and ePTFE) causes compliance mismatch due to their rigid nature unlike the native tissue [60]. Therefore, it is crucial to use biomaterials closely mimicking the natural ECM of the vessel wall. Compliance of a vascular graft can be measured by applying constant load on the graft while pressurizing internally (Fig. 3(b)). Then, the dimensional changes can be recorded and processed to measure the compliance.

### Suture retention

Suture is an essential surgical parameter in transplantation of artificial vascular grafts into the human body. Therefore, the transplanted graft should have enough strength to withstand the tensile load of the sutures without failure [61]. This is defined as the suture retention strength. To measure this strength, the prepared graft is cut from the middle. Then, they are sutured and pulled at a constant rate until rupture (Fig. 3(c)). The maximum tensile force is the suture retention strength.

### Endothelialization

The absence of endothelial layer in vascular devices may lead to health complications due to side effects such as thrombosis [62]. Thus, the formation of this specialized layer (the endothelium) is a fundamental step after transplantation for successful vascular regeneration. Since the endothelium is composed of ECs, the surface topography of the graft should promote cell adhesion and migration [63, 64]. Therefore, the surface chemistry of the vascular graft has an important role in the formation of the endothelium. Some of the most widely used methods to functionalize the vascular graft surface are listed in Table 2.

**Table 2:** Examples of surface modification methods for vascular grafts

**Surface modification method**	**Factor**	**Graft material**	**Results**	**Ref.**
Plasma treatment	Oxygen plasma	Polycaprolactone (PCL)	- Dense cellular infiltration	[65]
	Plasma immersion ion implantation	e-PTFE	- Rapid endothelialization - Suppressed early thrombosis	[66]
	Radio frequency glow discharges (RFGD)	Polyvinyl alcohol (PVA)	- Induce endothelialization	[67]
Immobilization	MSC-derived small extracellular vesicles (sEVs)	PCL	- Reduced thrombus formation - Enhanced endothelium formation	[68]
	Heparin/cell-adhesive peptides	Polyurethane (PU)	- Reduced platelet adhesion - Enhanced endothelial cell attachment	[69]
	Vascular endothelial growth factor (VEGF)	Poly(L-lactide-co-ε-caprolactone) (PLCL)	- Enhanced endothelial cell proliferation - Enhanced endothelial cell migration	[70]
	Fibronectin and stromal cell derived factor 1 alpha (FN-SDF-1α)	Dacron	- Early cell attraction - Improved endothelial coverage	[71]
Coating	Immobilized herapin	e-PTFE	- Reduced platelet adhesion - Decreased smooth muscle cell proliferation	[72]
	Gelatin	PCL	- Promote endothelialization	[73]

## Vascular tissue engineering

The intention of VTE is to guide cells to grow and mature into a functional blood vessel while the implanted graft is completely dissolved. 3D cell-printing is an emerging technology used in the field of tissue engineering capable of developing complex structures in high resolution mimicking the native environment of tissues and organs. 3D cell-printing process starts with the formulation of the bioink which is extruded through a small diameter nozzle attached to a 3D printing system.

### Core technology: 3D cell-printing

**Fig. 4:**
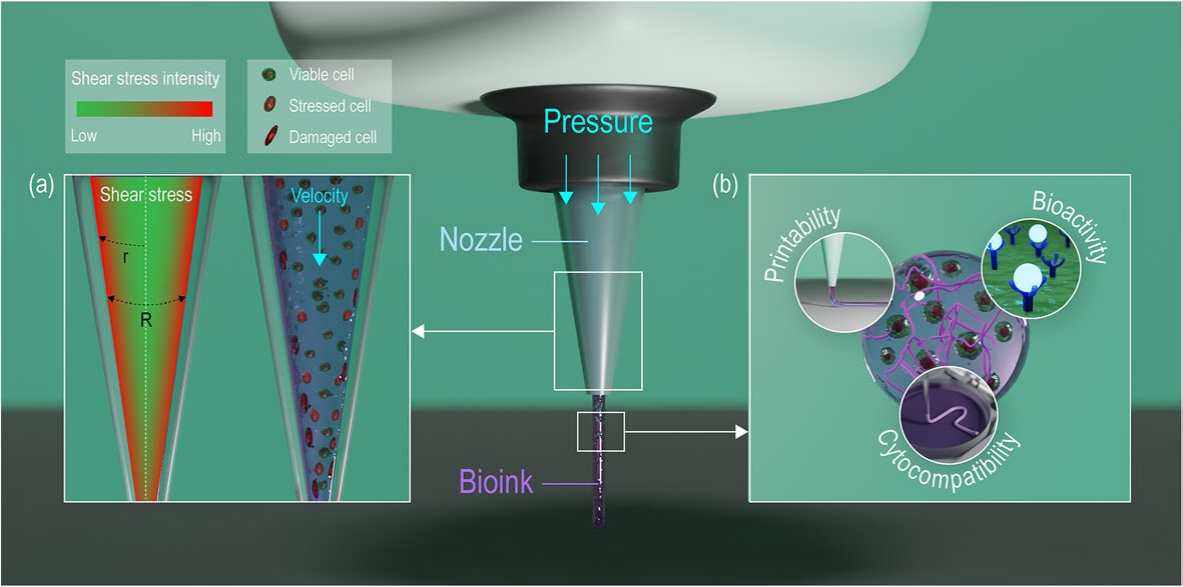
Conceptualized images showing the (**a**) effect of shear stress on cells in the bioink and (**b**) properties required for an ideal boink

3D cell-printing has revolutionized the field of TE in which carefully formulated bioink is extruded through the nozzle of a 3D cell-printing system to fabricate biological substitutes for tissue regeneration purposes. The replication of tissues and organs is a complex process, thereby 3D cell-printing systems should be highly accurate with high resolution. However, for high-resolution bioprinting small diameter nozzles are used in which the shear stress may cause low cell-viability. Prior to cell-printing, the printability of the bioink should be taken in consideration [74]. The printability is influenced by various parameters during the extrusion of the bioink. The flow behavior in the nozzle site can be described using the Herschel-Bulkley model, $\tau = {\tau }_{0}+ \text{{\rm K}}{\gamma }^{n}$, where $\tau$ defines shear stress, ${\tau }_{0}$ defines yield stress, $\text{{\rm K}}$ defines consistency index, $\gamma$ defines shear rate, and $n$ defines shear-thinning parameter [75, 76]. As seen in the Herschel-Bulkley model, shear stress is a function of shear rate. The fact that shear stress increases the risk of damaging the cells within the bioink is ubiquitous in the cell-printing world. The shear rate occurring in the nozzle can be defined as ${\gamma }^{n}= {\left[{V}_{2}{R}_{2}^{2}/\left(\frac{n}{3n+1}\right)\left({R}_{2}^{\frac{3n+1}{n}}\right)\right]}^{n}r$, where ${V}_{2}$ defines the velocity of the extruded bioink, ${R}_{2}$ defines the inner radius of the nozzle, and $r$ defines the radial distance from the axis of the nozzle [77]. The shear rate increases as the radial distance from the axis of the nozzle increases meaning that the shear stress also increases. The shear stress is greater in the area closer to the wall of the nozzle causing the greatest cell damage which can lead to cell death (Fig. 4(a)).

### Core resource: Bioink

Bioink is the core resource composed of printable biomaterials, viable cells, and other biological components essential for 3D cell-printing. Bioink should fulfill the physiological and physiochemical requirements associated with the printing process and the activities of the cells. Cell-friendly properties including biocompatibility, cytocompatibility, and bioactivity of the material are factors essential for obtaining high cell viability. Furthermore, rheological properties of the biomaterial play an important role not only in the viability of cells but also in the printability of cell-printing process. In short, an ideal bioink should be highly printable with high shape fidelity, protect cells from mechanical stress to maintain high cell viability, and provide biological cues to direct cellular activities (Fig. 4(b)). Some of the widely used biomaterials are listed in Table 3.

**Table 3:** Examples of natural biomaterials used in VTE

**Natural Polymer**	**Origin**	**Characteristics**	**Limitations**	**Ref.**
Alginate	Brown algae	- Rapid gelation - Economical	- Low cell adhesion	[78–80]
Chitosan	Arthropods Fungi	- Soluble in acidic media - Gelation at physiological temperatures	- Slow gelation rate	[81–84]
Collagen	Porcine Bovine Murine	- Most abundant protein in the body - Excellent bioactivities	- Low viscosity	[41, 85, 86]
Fibrin	Plasma proteins	- Major component of the blood clot	- Poor shape fidelity	[87–89]
Gelatin	Porcine Bovine Murine	- Denaturized collagen - Low antigenicity	- Liquification at physiological temperatures	[73, 90]
Hyaluronic acid	Bacteria	- Structural simplicity - Receptor-ligand interactions	- Lack of gelation abilities	[91, 92]

The key rheological parameters with the greatest influence on the cell-printing process are viscosity, yield stress, shear thinning, and viscoelasticity [93–95]. Viscosity is referred to the resistance to flow of a fluid under the application of stress. In general, highly viscous materials provide greater printability. However, increased viscosity causes increased shear stress in the printing nozzle which may negatively influence the viable cells in the bioink. On the contrary, low viscosity results in decreased printability leading to a poor shape maintenance after the printing process. Therefore, shear thinning behavior is important in extrusion-based printing systems. At rest, the molecular chains of the biomaterial are entangled and randomly oriented. When exposed to shear stress, the chains disentangle and orient along the shear flow. This behavior is called shear thinning, a phenomenon in which the viscosity decreases under shear stress [96]. Shear thinning behavior allows the ease of extrusion without harming the cells. Moreover, the decreased shear rate after the extrusion causes a rise in viscosity contributing to the shape preservation of the printed structure. During the extrusion through a nozzle, the bioink undergo viscous flow and elastic shape retention. This property is known as the viscoelasticity which can be determined using the storage modulus (G′) and the loss modulus (G″) [95]. G′ indicates the measurement of the energy stored elastically during deformation and G″ indicates the measurement of energy dissipated by the biomaterial. The G″/G′ ratio is defined as the loss tangent (tan(d)), which determines the state of the biomaterial. Greater values of tan(d) attribute to the extrusion uniformity, while lower values impact the shape fidelity. It is often overlooked the fact that cells within the bioink may alter the rheological properties [97–100]. Since cells occupy a certain volume, they can act as an obstacle hindering the crosslinking efficiency and chemical events. Therefore, the number of cells used should be considered.

## 3D cell-printing in vascular tissue regeneration

The anatomy and physiological conditions of tissues and organs differ from patient to patient. Therefore, patient-specific vascular grafts are highly demanded to prevent possible side effects after the transplantation. Using 3D cell-printing technology, tailor-made vascular grafts can be manufactured mimicking the native structure of the blood vessel. Besides graft production, 3D cell-printing can be utilized in other applications including vessel-on-a-chip.

### Efforts towards ideal tissue engineered vascular grafts

The first attempt was in 1986 where a multilayered tube was developed using collagen and a Dacron mesh [101]. Three types of cells (bovine aortic ECs, SMCs, and adventitial fibroblasts) were used to mimic the tri-layered structure of the blood vessel. The Dacron mesh was needed to compensate the weak strength of collagen. Unfortunately, despite the reinforcement, in vivo implantation was not possible due to low burst strength. In 1999, poly(glycolic acid) (PGA), a semi-crystalline synthetic polymer, and SMCs were used to develop a tissue engineered arteries [102]. A bioreactor with the ability to apply pulsatile radial stress improved the mechanical strength of the vascular structure. However, some challenges remained including polymer remnants after the implantation and lack of mature elastin on the developed vascular graft. Since then, various techniques have been employed to develop implantable grafts for vascular tissue regeneration.

### Cell-printed vascular grafts and other

**Fig. 5:**
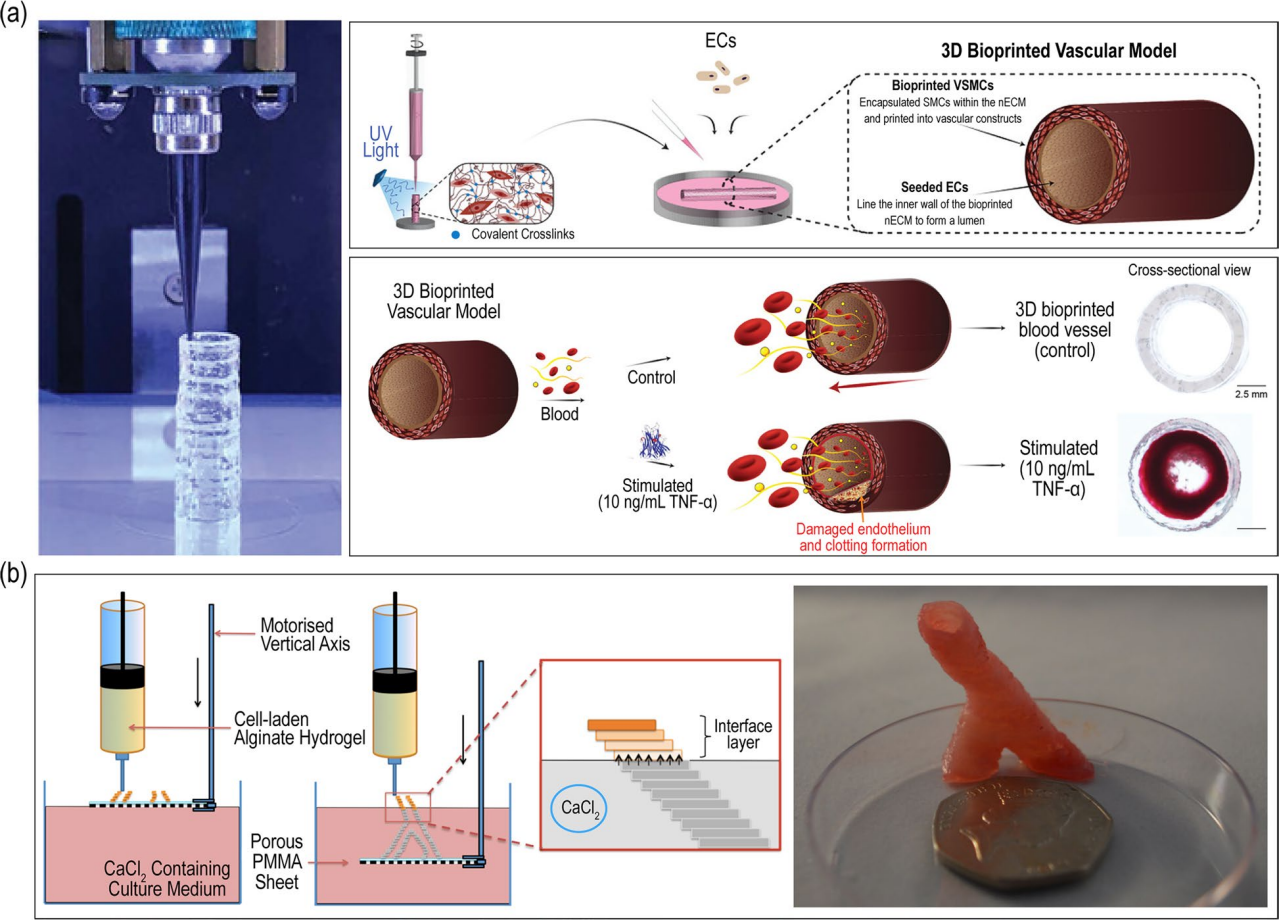
Fabrication of tubular vascular grafts using a layer-by-layer extrusion-based 3D cell-printing technique. Images showing production of (**a**) two-layered tubular structure for thrombo-inflammatory studies and (**b**) branched vascular structure. Reproduced from [103, 104] with permission from Wiley–VCH Copyright 2021 and IOP Publishing Copyright 2015

Various approaches towards extrusion-based 3D cell-printing of vascular grafts have been proposed. A simple method is to print a structure in a layer-by-layer manner. Gold et al. attempted to build a free-standing cylindrical vascular structure composed of gelatin methacryloyl (GelMA), polyethylene(glycol)diacrylate (PEGDA), and nanosilicates (nSi) using an extrusion-based cell-printing system (Fig. 5(a)) [103]. The cell-printed structure consisted of vascular smooth muscle cells (vSMCs) was stabilized through a subsequent crosslinking via UV light. Afterwards, ECs were seeded in the core region of the structure to replicate the natural structure of the blood vessel. Both cell types in the printed structure showed high rate of viable cells and phenotypic maintenance over time. Furthermore, cell-to-cell interaction was studied by assessing the EC barrier disruption and permeability to the underlying layer in a stimulated and healthy group. As a result, this vascular replica was able to mimic the in vivo thrombo-inflammatory responses. As another example, Tabriz et al. designed a branched vascular structure using a modified bioprinting technique in which the printing stage was able to displace in the z-direction [104]. As seen in Fig. 5(b), the cell-laden alginate bioink was crosslinked by submerging the structure into a CaCl_2_ bath by lowering the stage. To extend long term integrity, the printed structure was further crosslinked in barium chloride.

**Fig. 6:**
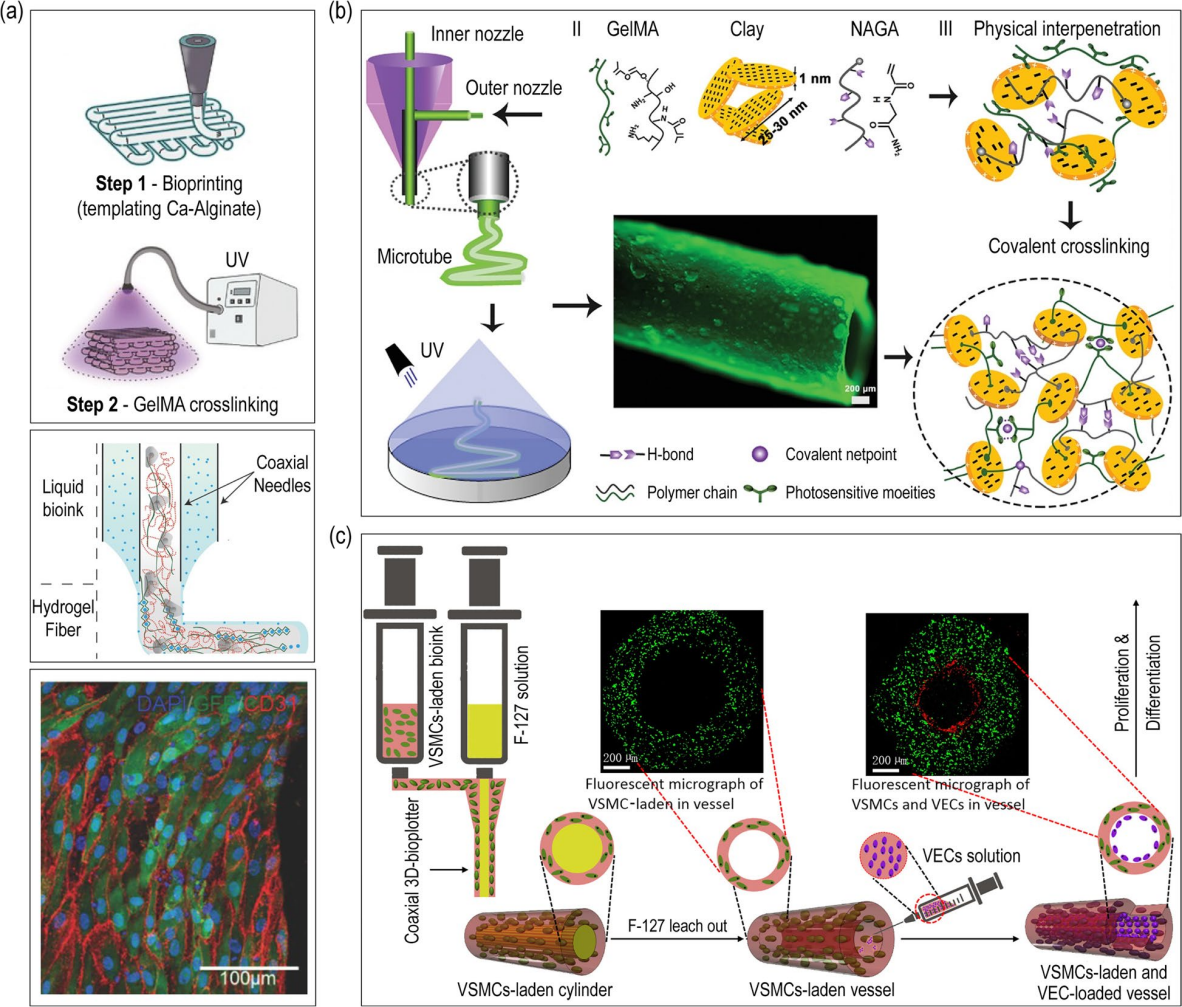
Coaxial extrusion 3D cell-printing of microtubes using (**a**) GelMA/alginate, (**b**) nanoclay/N-acryloyl glycinamide (NAGA)/GelMA, and (**c**) GelMA/PEGDA/alginate/lyase Reproduced from [105–107] with permission from Wiley–VCH Copyright 2015, 2020, and American Chemical Society Copyright 2020

One of the most efficient and promising extrusion-based cell-printing strategy in producing tubular structures is the core-shell printing method. This method allows the use of multiple bioinks with different cell types to simulate the blood vessel structure. Colosi and coworkers developed a structure composed of tubular struts laden with human umbilical vein endothelial cells (HUVECs) [105]. A coaxial nozzle attached to a 3D bioprinter was employed where GelMA, alginate, and photoinitiator was extruded through the inner nozzle and CaCl_2_ solution was flown through the outer nozzle to ionically crosslink the alginate chains (Fig. 6(a)). Afterwards, the printed structure was UV crosslinked to stabilize the GelMA prepolymer. The bioprinted HUVECs colonized at the edge of the struts forming a vessel-like structure after 10 days. Moreover, the cells were uniaxially aligned forming a monolayer mimicking the structure of the native blood vessel. However, vascular grafts fabricated in the above-mentioned examples lack the mechanical properties which do not match the standards for burst pressure and suture retention strength.

In another study, a high-strength small diameter vascular graft was manufactured using a core-shell extrusion printing system [106]. The biomaterials used are nanoclay, N-acryloyl glycinamide (NAGA), and GelMA (Fig. 6(b)). The amount of nanoclay was fixed at 100 mg, while the amount of NAGA and GelMA were varied. The mechanical strength was dependent on the ratio between NAGA and GelMA. Greater content of NAGA resulted in enhanced mechanical strength. Also, greater burst pressure and suture retention strength was achieved compared to the native tissue. A burst pressure of ≈ 1500–2500 mmHg was achieved which was in the range of the standard for autologous vascular graft. Moreover, the suture retention strength of the fabricated graft (≈ 280 gf) was significantly greater than that of the human saphenous vein (196 ± 2 gf) [108]. Besides physical properties, the designed tubular graft showed outstanding biocompability. In a study of Zhou et al., a small-diameter blood vessel composed of two different cell layers was fabricated using a core-shell printing system [107]. To obtain the lumen structure, F-127 was used to leach out the core of the printed vessel. In the shell region, vSMCs were embedded in the bioink composed of GelMA/PEGDA/alginate/lyase. For the stabilization of the printed structure, alginate was ionically crosslinked using CaCl_2_ solution and GelMA/PEGDA was photo-crosslinked using a UV laser. Finally, vascular endothelial cells (vECs) encapsulated in gelatin were injected in the core region. This process is shown in Fig. 6(c). The cell-laden structure was perfusable under various conditions (flow velocity, flow viscosity, and temperature). The fabricated structure was considered to have similar elasticity properties (compliance) of real blood vessels under various physiological conditions. Furthermore, lyase in the bioink accelerated the degradation of alginate which provided space for the cells to proliferate.

A similar coaxial bioextrusion method was used to fabricate an in vitro vasculature model [109]. Vasculature is an essential part in organ-on-a-chip development for replacing animal testing and studying the human body. First, a polymeric chamber was printed using poly(ethylene-co-vinyl acetate) (PEVA) before cell-printing the vessel composed of alginate, vascular-tissue-derived extracellular matrix (vECM), and HUVECs. The next step is the maturation of the cell-printed vessel where an endothelial monolayer is formed. This vascular model can be used for studying the pathological changes during the process of inflammatory diseases.

### In vivo applications of cell-printed vascular grafts

**Fig. 7:**
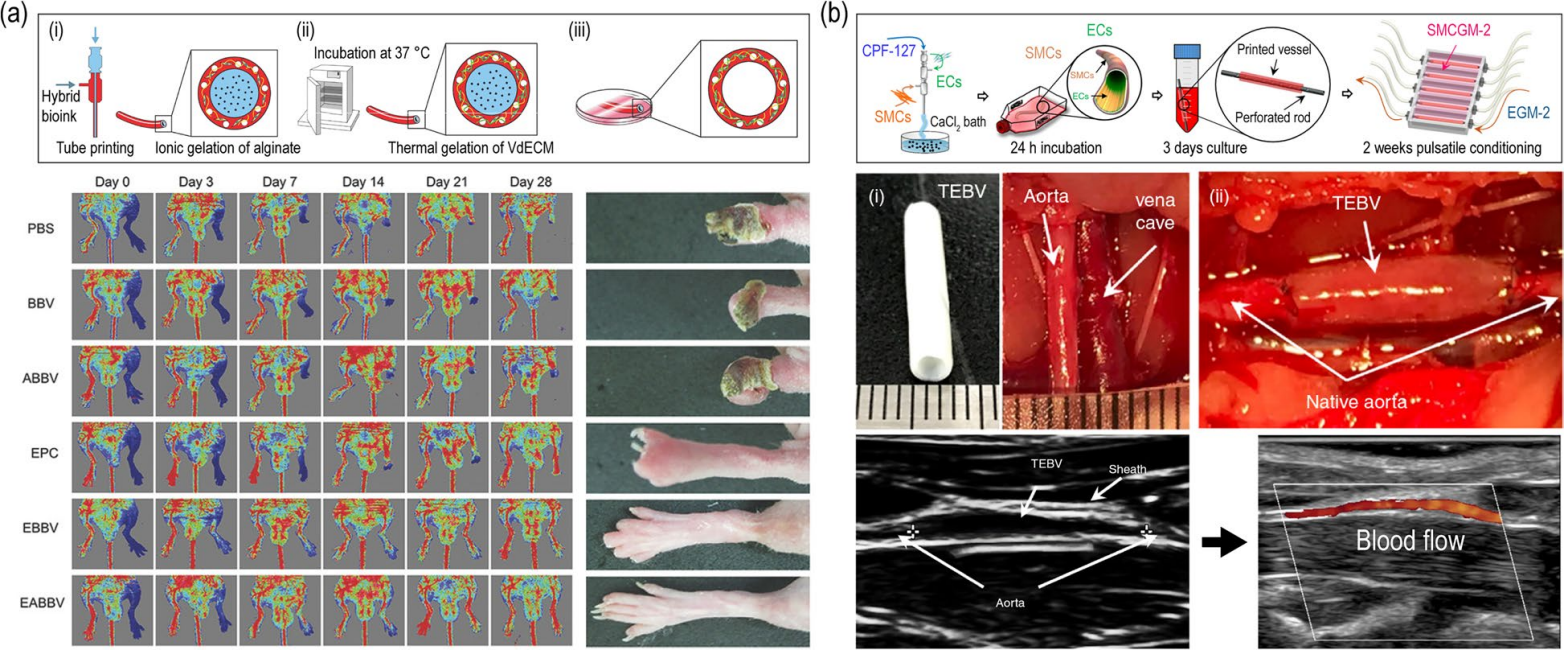
Clinical applications of tissue engineered vascular grafts using (**a**) coaxial and (**b**) triple-coaxial cell printing techniques. Reproduced from [110, 111] with permission from Wiley–VCH Copyright 2017 and AIP Publishing Copyright 2019

Gao et al. engineered a tubular structure composed of atorvastatin-loaded poly(lactic-co-glycolic) acid (PLGA) microspheres/vECM/alginate with endothelial progenitor cells (EPCs) [110]. In this process, a coaxial cell-printing method was used where pluronic F-127 was used in the core region as a sacrificial material to create the tubular structure (Fig. 7(a)). This vascular structure was transplanted into a nude mice hind limb to study the therapeutic effect on the ischemic disease. Reduced limb loss, foot necrosis, and toe loss was observed in the group transplanted with the fabricated vascular structure. In addition, increased neovascularization was discovered at the injury site when transplanted with the cell-printed structure. This research group also attempted to mimic the structure of the natural blood vessel using a triple-coaxial cell printing system as seen in Fig. 7(b) [111]. The bioink used in their study was composed of alginate and vECM. To create the outer layer, vSMCs were encapsulated in the bioink. For the inner layer, vECs were embedded in the bioink. The tubular structure was formed by leaching out the PF-127 in the core. After culturing the cell-laden structure in a bioreactor system, it was implanted in rat abdominal aorta and observed for three weeks. As a result, the tissue engineered vascular graft showed promising results including great patency, well-retained endothelium, matured smooth muscles, and integration with host tissues.

## Requirements for commercialization

One of the most significant hurdles for commercialization of tissue engineered vascular graft is to get the approval by governmental organizations such as Food and Drug Administration (FDA). One of the requirements is that the vascular graft should be fabricated under good manufacturing practice (GMP) conditions. GMP involves the manufacturing and management of the medicinal products according to the quality standards for product approval. Therefore, the 3D bioprinting systems should be GMP grade and placed in GMP facilities operated by trained authorities. Moreover, the biomaterials used to fabricate the grafts should get approved or be on the list of approved materials. After the production process, the vascular grafts should pass various safety tests including biocompatibility test (ISO 10,993), chemical-physical and performance test (ISO 7198:2007, ISO 25539-1:2010, and ISO 15676:2005), and pre-clinical test (Good Laboratory Practice (GLP)). Finally, the last step prior to approval is the clinical trial (ISO 14,155). This whole approval process might be enduring and costly. The estimated time for FDA approval when it comes to a tissue engineered vascular graft is approximately 10 years.

## Conclusions and future perspectives

As mentioned in this review, autografts remain the gold standard for blood vessel regeneration. However, shortage in supply has forced to search for an alternative. As a solution, 3D cell-printing technology has been introduced capable of producing vascular grafts using cell encapsulated bioink. An ideal vascular graft should closely mimic the structure and function of the natural blood vessel. For this purpose, the cell-printing system and bioink should be optimized. The printing system should be able to simulate the three-layered structure of the blood vessels. Moreover, the bioink should protect cells against the shear stress inside the nozzle and provide appropriate environment to guide the cells. For clinical applications, the fabricated vascular graft should withstand the blood pressure, match the compliance with that of the host tissue, and bear the tensile load of the sutures during implantation. Despite of the advances in 3D cell-printing technology, there are still some hurdles to overcome.

One of the most critical challenges is to enhance the mechanical strength of the biomaterials used for cell-printing. In general, bioinks are composed of hydrogels to encapsulate viable cells harmlessly. However, one limitation of these vascular structures is the mechanical properties which were not in the range of a native vessel. Another challenge is the multi-cell culture since various cells are embedded in the vascular graft. Therefore, the multi-cell culture should be optimized to provide proper environment and induce cell differentiation/maturation. In terms of commercialization, some practical challenges exist to overcome. Commercial-grade production process is far more complicated when cells are involved. First, good manufacturing practice (GMP) production facilities are required. Second, the shell life of the vascular graft should be above the minimum standard. Third, the maintenance & storage problems should be solved. To this end, solution to these limitations is needed to provide patients suffering from vascular diseases with commercially available products.

## Data Availability

All data generated or analyzed during this study are included in published article.

## Abbreviations

ePTFE: expanded poly(tetrafluoroethylene)
VTE: vascular tissue engineering
SMCs: smooth muscle cells
ECs: endothelial cells
ECM: extracellular matrix
G′: storage modulus
G″: loss modulus
PGA: poly(glycolic acid)
GelMA: gelatin methacryloyl
PEGDA: polyethylene(glycol)diacrylate
nSi: nanosilicates
HUVECs: human umbilical vein endothelial cells
vSMCs: vascular smooth muscle cells
NAGA: N-acryloyl glycinamide
vECS: vascular endothelial cells
PEVA: poly(ethylene-co-vinyl acetate)
vECM: vascular-tissue-derived extracellular matrix
PLGA: atorvastatin-loaded poly(lactic-co-glycolic) acid
EPCs: endothelial progenitor cells
GMP: good manufacturing practice
FDA: Food and Drug Administration
GLP: Good Laboratory Practice
